# Interleukin-7 expression by CAR-T cells improves CAR-T cell survival and efficacy in chordoma

**DOI:** 10.1007/s00262-024-03756-9

**Published:** 2024-08-02

**Authors:** Huantong Wu, Zhuofan Xu, Maoyang Qi, Penghao Liu, Boyan Zhang, Zhenglin Wang, Ge Chen, Xiaohai Liu, Junqi Liu, Wei Wei, Wanru Duan, Zan Chen

**Affiliations:** 1 Department of Neurosurgery, Xuanwu Hospital, Capital Medical University, Beijing, China; 2 Lab of Spinal Cord Injury and Functional Reconstruction, China International Neuroscience Institute (CHINA-INI), Beijing, China; 3 Department of Otorhinolaryngology Head and Neck Surgery, Xuanwu Hospital, Capital Medical University, Beijing, China

**Keywords:** B7–H3, Chimeric antigen receptor T cells, Chordoma, Interleukin-7

## Abstract

Chordoma is a rare bone tumor that frequently recurs after surgery, and the prognosis is poor with current treatments. This study aimed to identify potential novel immunotherapeutic targets for chordomas by identifying target proteins in clinical samples as well as tumor microenvironmental factors to enhance efficacy. Fourteen chordoma samples were analyzed by single-cell RNA sequencing, and B7–H3 and IL-7 were identified as potential targets and potentiators, respectively. B7–H3-targeted chimeric antigen receptor T (CAR-T) cells and B7–H3 CAR-T cells expressing IL-7 were synthesized and their anti-tumor activity evaluated in vitro, including in primary chordoma organoid models. The B7–H3 CAR-T/IL-7 therapy showed enhanced cytotoxicity and prolonged duration of action against tumor cells. Additionally, IL-7 modulated favorable subpopulations of cultured CAR-T cells, diminished immune checkpoint expression on T-cell surfaces, and enhanced T-cell functionality. The incorporation of IL-7 molecules into the B7–H3 CAR structure augmented CAR-T-cell function and improved CAR-T-cell efficacy, thus providing a novel dual therapeutic strategy for chordoma treatment.

**Supplementary Information:**

The online version contains supplementary material available at 10.1007/s00262-024-03756-9.

## Introduction

Chordoma, an exceedingly uncommon but locally aggressive bone cancer, has an annual incidence of roughly 0.8 per 1,000,000 individuals in both the US and Europe, equating to ~ 350 cases each year [1–5]. Although accounting for only 1–4% of all primary bone cancers, chordomas have a high local recurrence rate, causing local bone destruction, compression of surrounding structures, and a consequently poor prognosis for many patients (median survival 6–7 years) [6–8]. A favorable clinical outcome largely depends on the successful surgical removal of the tumor, but the effectiveness of surgery may be hindered by involvement of other anatomical structures caused by local spread after delays in the diagnosis due to the often the subtle and non-specific presenting symptoms [9, 10]. Given the ineffectiveness of conventional chemotherapy, particularly in early-stage patients, a combination of surgery and adjuvant radiotherapy is usually deployed to achieve long-term local tumor management and growth control [11, 12]. Nevertheless, effective therapies that better control growth and reduce recurrences are urgently needed.

Recent advances in single-cell RNA sequencing (scRNA-seq) technologies offer a valuable means to comprehensively profile and understand the regulatory networks, heterogeneity, and mechanisms of cancers, including chordomas [13]. B7–H3 is an immune checkpoint in the B7 family that interacts with checkpoint markers such as LAG-3 (lymphocyte activation gene-3) and PD-1 (programmed death-1). B7–H3 is overexpressed in many cancer types, so is an attractive immunotherapy target [14–18]. Chimeric antigen receptor T (CAR-T)-cell therapy has emerged as an efficacious therapeutic modality, but its application to solid tumors is hampered by the limited availability of target antigens, constrained tumor site infiltration, antigenic heterogeneity and depletion, and the existence of an immunosuppressive milieu within the tumor microenvironment (TME) [19]. Many strategies to augment CAR-T cells efficacy in solid tumors have been proposed, including the utilization of dual CAR designs with the ability to concurrently identify multiple antigens, the incorporation of oncolytic viruses, expression of cytokines or chemokines, and the eradication of other inhibitory elements within the microenvironment [20–22].

Human interleukin-7 (IL-7) is a pleiotropic immune cytokine that exerts both direct and indirect effects on anti-tumor activity and impacts the growth, survival, and differentiation of B and T cells [23–25]. Clinical data have consistently shown that IL-7 co-expression prolongs survival of CAR-T cells and improves their ability to ability to expand and kill tumors [26–28]. When exposed to IL-7, CAR-T cells persist and enhance anti-tumor activity in vivo [29, 30]. In addition, IL-7 can amplify initial T cells and anti-tumor activity with minimal side effects and good patient tolerance, providing an opportunity to use of IL-7 in tumor therapy [31–33].

Here, we describe the characteristics of chordoma using scRNA-seq to characterize tumor heterogeneity and identify rational therapeutic targets. Taking the pathological characteristics of the chordoma TME into account, we designed a CAR-T-cell preparation, B7–H3 CAR-T/IL-7, that not only recognizes the chordoma-specific antigen B7–H3 but also enhances the anti-tumor capacity of CAR-T cells by secreting IL-7. In this way, the therapy exerts dual therapeutic effects to overcome the limitations of current chordoma treatment.

## Methods

### Chordoma samples and healthy controls

This study included 14 patients who underwent surgical resection of chordoma at Xuanwu Hospital, Capital Medical University, Beijing, China, between July 2019 and April 2023. Patients who had received preoperative radiotherapy, chemotherapy, or other targeted therapies were excluded. Chordoma was diagnosed based on the 2013 World Health Organization classification of bone tumors, identifying each as classic chordoma. Immunohistochemical analysis confirmed strong TBXT (brachyury) expression in the chordoma cells of all patients. Fresh tumor samples were obtained intraoperatively. Every patient provided written informed consent, and the study protocol was approved by the Ethics Committee of Xuanwu Hospital, Capital Medical University (approval number: [2021]021). Single-cell transcriptome data of three human nucleus pulposus samples were obtained from the GEO database (GSE160756) as controls [34].

### Single-cell RNA sequencing

ScRNA-seq was performed using the Single Cell 3′ Library & Gel Bead Kit V2 (10 × Genomics, Pleasanton, CA), with cell suspensions processed on a Chromium Single Cell Controller and sequencing performed on an Illumina HiSeq X Ten (Illumina, San Diego, CA).

Data were processed and analyzed using Seurat (v3.2.0) in R (v4.0.2). Initial data were filtered according to two primary criteria: cells expressing at least 300 genes and genes expressed in at least three cells, coupled with a mitochondrial content threshold < 15%. This filtration process yielded 109,453 cells from 14 chordoma samples and 37,334 cells from the three nucleus pulposus samples. Seurat was also used for data normalization and integration to mitigate batch effects. An integrated expression matrix was generated, scaled, and subjected to principal component analysis (PCA) and UMAP visualization to delineate the cellular landscape. Cellular clusters were defined using Seurat, resulting in the identification of 12 distinct clusters. Cluster markers were identified, and cell types were annotated according to canonical marker genes, the CellMarker website, and the inferCNV package (v1.5.0) for guidance. The *TBXT* gene, characteristic of chordoma cells, served as a key marker in tumor cell annotation. For differential gene expression (DEG) analysis, the Wilcoxon rank-sum test was used to compare gene expression levels between identified cell clusters.

### Primary cell cultures

Chordoma tumor tissue was rinsed with 5-mL phosphate-buffered saline (PBS) in a 10-cm dish, followed by meticulous mincing using surgical scissors. Subsequently, tumors were cut into small fragments (1–3 mm^3^) using sterile scissors. Tissue was then digested with collagenase IV (200 U/mL) at 37 °C in a 5% CO_2_ environment, with vigorous shaking every 15 min, for 2 h. Following digestion, samples were filtered through 70-μm cell sieves into fresh 15-mL centrifuge tubes. The sample was obtained by centrifuging at 800 rpm for 3 min, and the supernatant was aspirated and discarded. A mixture of MammoCult medium and Matrigel at a 3:4 ratio was introduced. Subsequently, 10^5^ isolated primary chordoma cells were incorporated into a 70-μl suspension and placed in a single circular formation at the periphery of a 24-well plate. Following this, 1 ml of MammoCult medium was added, and the plate incubated.

### Cell line cultures

Human chordoma cell lines UCH2 and JHC-7 were procured from the ATCC. The human embryonic kidney cell line HEK392T and the human colon cancer cell line HT1080 were obtained from Wuhan Procell Biotech Company (Wuhan, China) for lentivirus preparation and titer assays. JHC-7 cells were cultured in DMEM/F12 medium supplemented with 10% FBS at 37 °C in a humidified atmosphere containing 5% CO_2_. The UCH2 cell line was cultured in a mixture of IMDM and RPMI 1640 (4:1) with the addition of 20% FBS. HEK293T and HT-1080 cell lines were maintained in DMEM supplemented with 10% FBS and 1-mM GlutaMax at 37 °C. Prior to experimentation, all cell lines were confirmed to be *Mycoplasma* free using the Rapid Mycoplasma Test kit (Cellapybio, Beijing, China) according to the manufacturer’s instructions. No *Mycoplasma* was detected in any cell line.

### *CAR* design

The CAR structure is described in our previous work [35]. The CD5 scFv was changed into B7–H3 scFv. This CAR structure contains a hinge domain, a transmembrane domain, a co-stimulatory domain, and a CD3ζ signaling domain. The B7–H3 CAR construct used the 4-1BB co-stimulatory domain with a CD8 transmembrane domain (BB.z). The sequence encoding *IL7* was designed by our laboratory, and the B7–H3 and IL-7 structures were combined with P2A.

### *CAR*-T-cell production

Peripheral blood mononuclear cells (PBMCs) were isolated by Ficoll-Paque gradient centrifugation. CD3^+^ T cells were purified and stimulated using CD3/CD28 magnetic beads (Gibco, Thermo Fisher Scientific, Waltham, MA) at a ratio of 1:2. T cells were then sorted using magnetic beads placed in Ultra-Low Attachment Surface 6 wells at a density of 1.5 × 10^6^/mL for activation in X-VIVO 15 medium (Lonza, Basel, Switzerland) supplemented with 500 IU/mL IL-2 (XinLuoEr, Shanghai, China), 80 ng/mL IL-7 (Jin’an, China), 20 ng/mL IL-15 (Jin’an, China), and 20 ng/mL IL-21 (Jin’an, China). Following a 10-day cell culture period, transduction efficiency was assessed.

### Flow cytometry

The following anti-human antibodies were procured from BioLegend (San Diego, CA, USA): CD3, CD4, CD8, CD197 (CCR7), CD45RO, B7H3, PD-1, LAG3 (FITC, PE, PerCP, APC), and streptavidin-PE. Biotin-labeled Fab was acquired from Abcam (Cambridge, UK) for the quantification of CAR-positive T cells, and the presence of biotin-Fab was detected through the addition of streptavidin-PE. Flow cytometry analysis was conducted using FACSCalibur (BD Biosciences, San Jose, CA), and the obtained data were analyzed using FlowJo v10.0.

### In vitro cytotoxicity assay

CAR-T cells and chordoma cell lines were co-cultured at different effector-to-tumor (E/T) ratios. In the transient cytotoxicity assay, the co-culture supernatant from various E/T ratios was collected after 20 h of cultivation. In the multi-round cytotoxic assay, effector cells were co-cultured with target cells at an E/T ratio of 4:1, with equal amounts of target cells added every other day. The supernatant samples were collected every 48 h, and the initial number of tumor cells was reintroduced into the co-culture system to re-challenge the CAR-T cells. Supernatant detection was conducted according to the Non-Radioactive Cytotoxicity kit (Promega, USA) instructions. The results were subsequently calculated following the manufacturer’s instructions.

### Cytokine release assay

Cytotoxic cytokines IL-2, IFN-γ, IL-6, IL-7, and TNF-*α* were tested by ELISA in vitro. Supernatants were collected from samples and centrifuged at 800 × g for 15 min at 4 °C. Cytokines were quantified using ELISA kits (Neobioscience, Shenzhen, China) following the manufacturer’s instructions. Briefly, samples were collected and centrifuged at 800 × g for 15 min at 4 °C, and the assay was performed following the manufacturer’s instructions. ELISA data were acquired using Varioskan Flash (Thermo Fisher Scientific, Waltham, MA).

### Statistical analysis

Data are presented as mean ± standard deviation (SD). Statistical analyses were performed using Prism (GraphPad Software, La Jolla, CA). Student’s *t*-test was used as a two-sided paired test with 95% confidence intervals (CI) for comparisons between two groups. For comparisons involving three or more groups, two-way analysis of variance (ANOVA) was conducted with Dunnett’s multiple comparisons test. Results with a p-value less than 0.05 were deemed statistically significant.

## Results

### Single-cell transcriptomic analysis identifies CD276 (B7–H3) as a potential target in chordoma

Fourteen tumor samples and three nucleus pulposus samples were subjected to scRNA-seq profiling. Chordoma samples were obtained from patients aged between 17 and 79 years at the following sites: sacrum (n = 5), spine (n = 7), and skull base (n = 2). Details of the patient characteristics are shown in Supplementary Table 1.

**Fig. 1:**
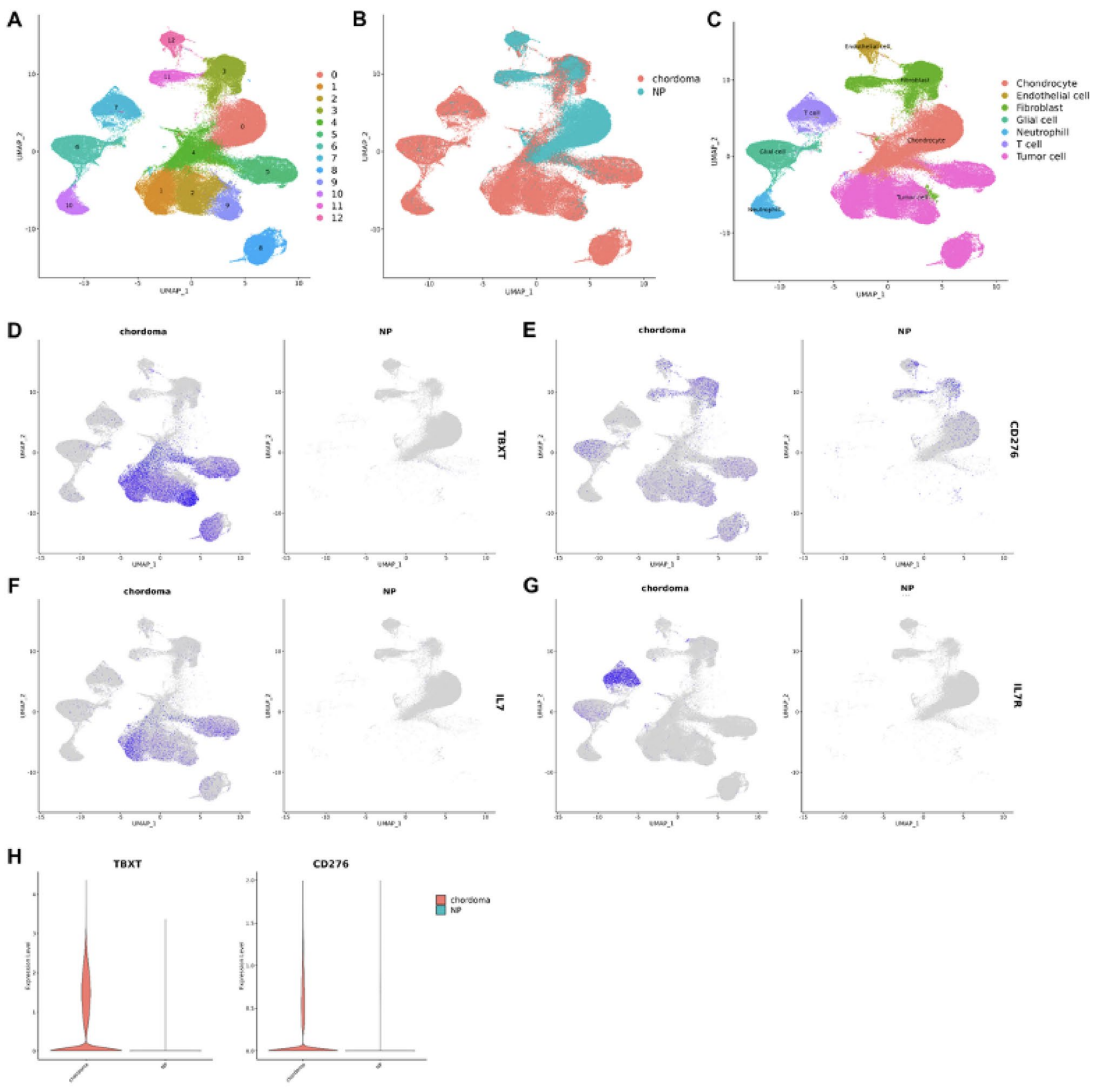
Single-cell transcriptomic analysis of chordoma and control samples. **A–C** UMAP clustering, grouping, and cell type identification for chordoma and NP cells. **D–E** Expression of *TBXT* and *CD276* was predominantly seen in tumor cells. **F–G** Expression of *IL7* was seen in tumor cells, while expression of *IL7R* was seen in T cells; **H** Violin plot of *TBXT* and *CD276* expression in chordoma and NP cells

After quality control, 109,453 cells from 14 chordoma samples (chordoma group) and 37,334 cells from the three nucleus pulposus samples (NP group) were included in the analysis. After normalization of gene expression and PCA, cells from the two groups were divided into 12 clusters using the UMAP method (Fig. 1A and B). Clusters were further assigned to seven known cell lineages: chondrocytes, endothelial cells, fibroblasts, glial cells, neutrophils, T cells, and tumor cells (Fig. 1C). Differential expression analysis identified several genes enriched in the tumor cells compared with chondrocytes from the NP group (Supplementary Fig. S1). Among them, we focused on *CD276*, since the *CD276*-encoded protein, B7–H3, is overexpressed in several other cancers, is an immunoregulatory protein, and is already targeted by CAR-T cells. Expression of *TBXT* (as a tumor cell marker) and *CD276* is shown in Fig. 1D and E. *CD276* expression was high in chordoma cells compared with NP cells (Fig. 1H), and although predominantly expressed in tumor cells, *CD276* was also present in fibroblasts and glial cells.

We also investigated the immune microenvironment, finding high expression of *IL7* in tumor cells and high *IL7R* expression in tumor-infiltrating T cells (Fig. 1F and G), suggesting that immune interactions also play a role in local tumor invasion and progression.

### *CAR* structure and expression of *CAR*-positive T cells

**Fig. 2:**
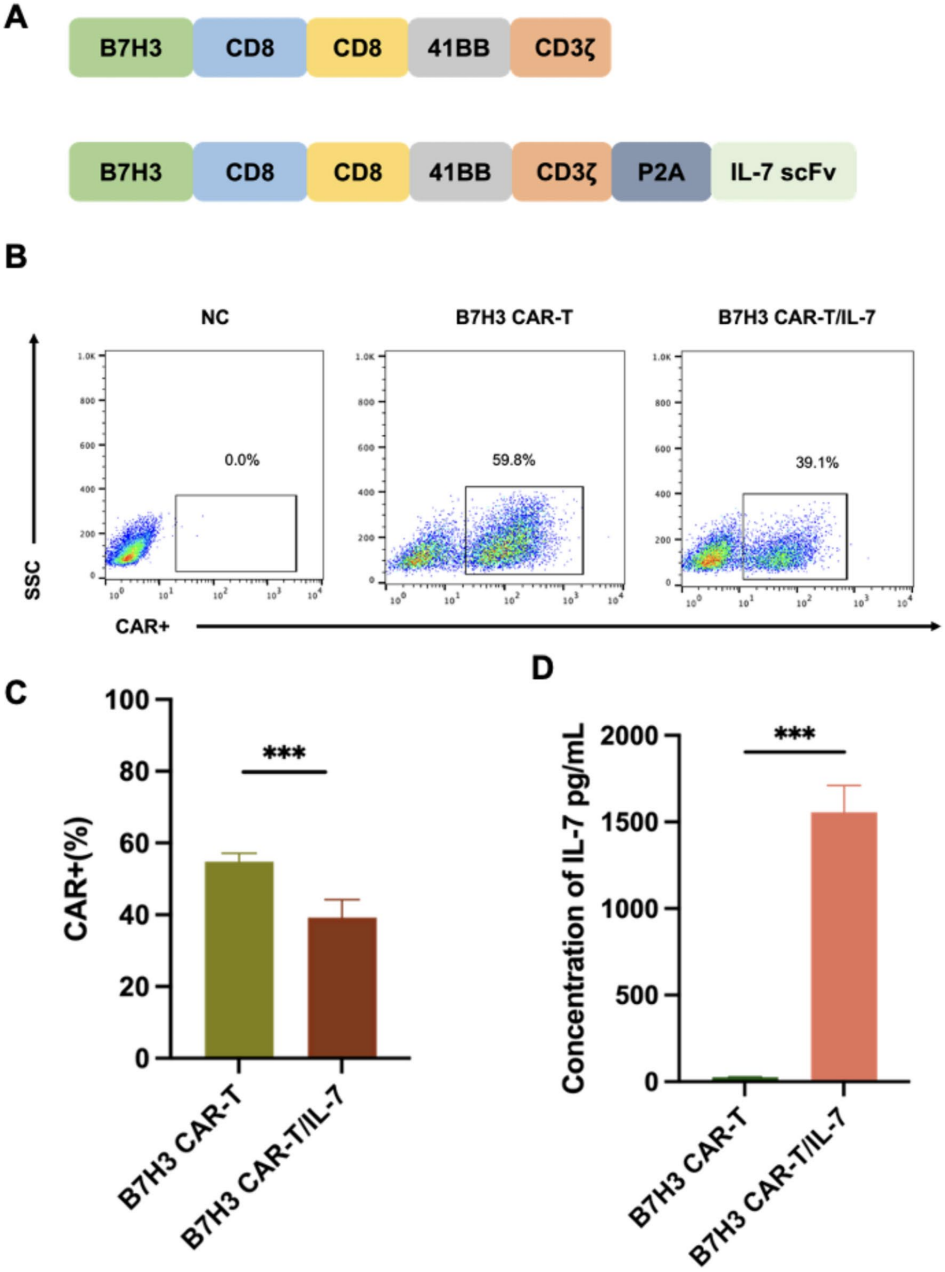
Single-cell transcriptomic analysis of chordoma and control samples. **A** Schematic of the construction of B7–H3-specific CARs. **B** Representative flow cytometry analysis showing CAR expression on T cells transduced with B7–H3 or B7–H3/IL-7 on day 7. **C** CAR expression on T cells transduced with B7–H3 or B7–H3/IL-7 on day 7. **D** Competitive binding of CAR-T-cell supernatants to IL-7. Data shown are mean ± SD (n = 3). **p < 0.01 and ***p < 0.001 (two-way ANOVA)

Two CARs incorporating 4-1BB co-stimulatory domains and targeting B7–H3 were generated: B7–H3 CAR and B7–H3 CAR combined with IL-7 (Fig. 2A). B7–H3 CARs and B7–H3 CAR-T/IL-7 were expressed on the surface of transduced T cells (Fig. 2B). B7–H3 CAR-T cells were significantly more frequent than B7–H3 CAR-T/IL-7 cells in the CAR^+−^ population (p < 0.01) (Fig. 2C). To examine IL-7 expression by T cells transfected with B7–H3 CAR and B7–H3 CAR-T/IL-7 constructs, IL-7 levels in the culture supernatant of CAR-T cells were detected by ELISA (Fig. 2D), which confirmed expression of IL-7 by B7–H3 CAR-T/IL-7 cells.

### Enhanced anti-tumor cytotoxicity of B7–H3 *CAR*-T/IL-7 cells in vitro

**Fig. 3:**
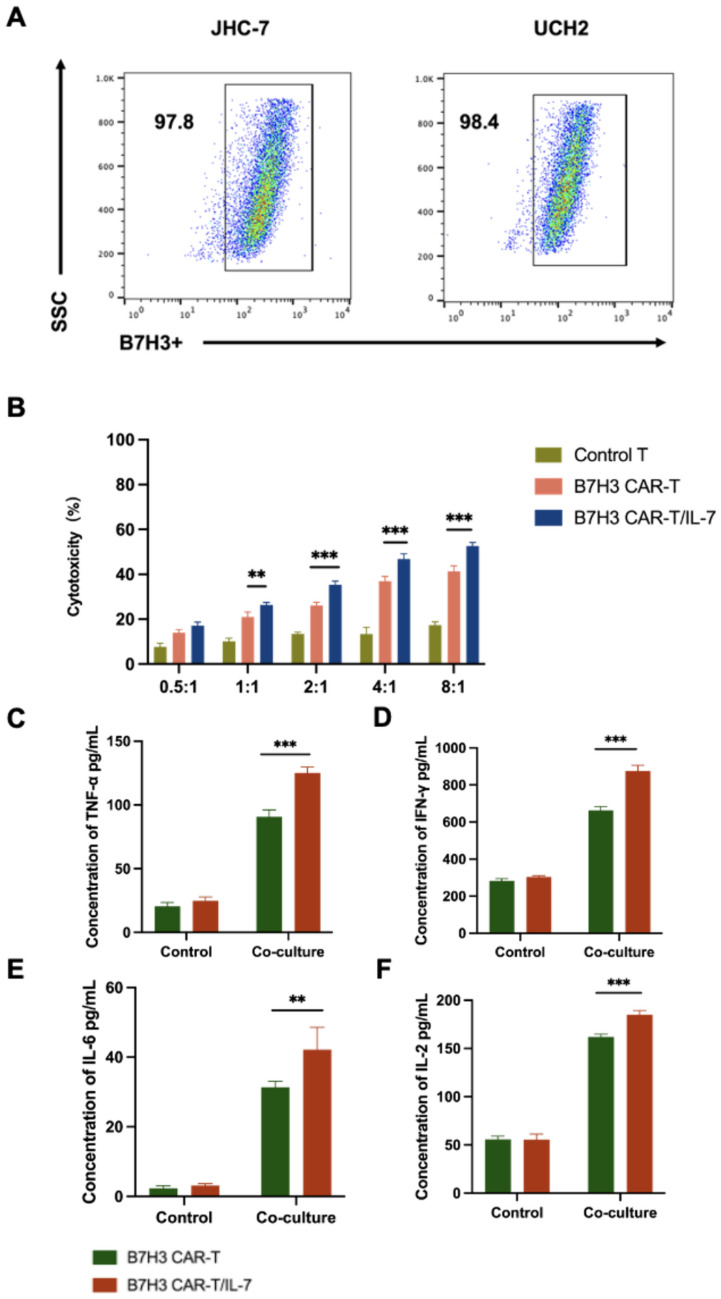
B7–H3 CAR-T/IL-7 cells enhance anti-tumor cytotoxicity in vitro*.* A Expression of surface B7–H3 on two chordoma cell lines, JHC-7 and UCH2. **B** B7–H3 CAR-T and B7–H3 CAR-T/IL-7 cells were co-cultured with JHC-7, a chordoma cell line, at different E/T ratios (from 0.5:1 to 8:1). Cytotoxicity was measured by the LDH release assay after a 20-h incubation. **C-F** ELISA data showing the quantification of cytokines (IL-6, IFN-γ, IL-2, and TNF-*α*) in the supernatants after B7–H3 CAR-T and B7–H3 CAR-T/IL-7 cells were co-cultured with JHC-7 at an E:T ratio of 8:1 for 20 h. Data shown are mean ± SD (n = 3). *p < 0.05, **p < 0.01, ***p < 0.001, and ****p < 0.0001 (two-way ANOVA). Not significant (ns)

We next assessed the activation and killing effects of our CAR-T cells on chordoma cells expressing B7–H3 using the JHC-7 and UCH2 chordoma cell lines. Both cell lines (JHC-7 and UCH2) highly expressed B7–H3 targets on the surface, as assessed by FACS (Fig. 3A). Control, B7–H3 CAR-T, and B7–H3 CAR-T/IL-7 cells were incubated with JHC-7 and UCH2 cells at different effector-to-target (E/T) ratios. B7–H3 CAR-T and B7–H3 CAR-T/IL-7 cells both showed cytotoxicity against JHC-7 cells that were positively correlated with E/T ratios. Cells were quantified based on the number of positive CAR-T cells. Remarkably, B7–H3 CAR-T/IL-7 cells exhibited greater cytotoxicity than B7–H3 CAR-T cells (Fig. 3B). Incubation of the two CAR-T cells with JHC-7 cells at an 8:1 ratio resulted in the release of pro-inflammatory cytokines including interferon-gamma (IFN-γ), tumor necrosis factor-alpha (TNF-*α*), IL-2, and IL-6 (Fig. 3C, D, E, and F). B7–H3 CAR-T/IL-7 cells exhibited greater release of all four cytokines compared with B7–H3 CAR-T cells. Similarly, when incubated with UCH2 cells, B7–H3 CAR-T/IL-7 cells significantly enhanced tumor-killing activity and promoted the release of pro-inflammatory cytokines (Supplementary Fig. S2).

### IL‑7 enhances persistence of *CAR*‑T cells against tumor cells in vitro

**Fig. 4:**
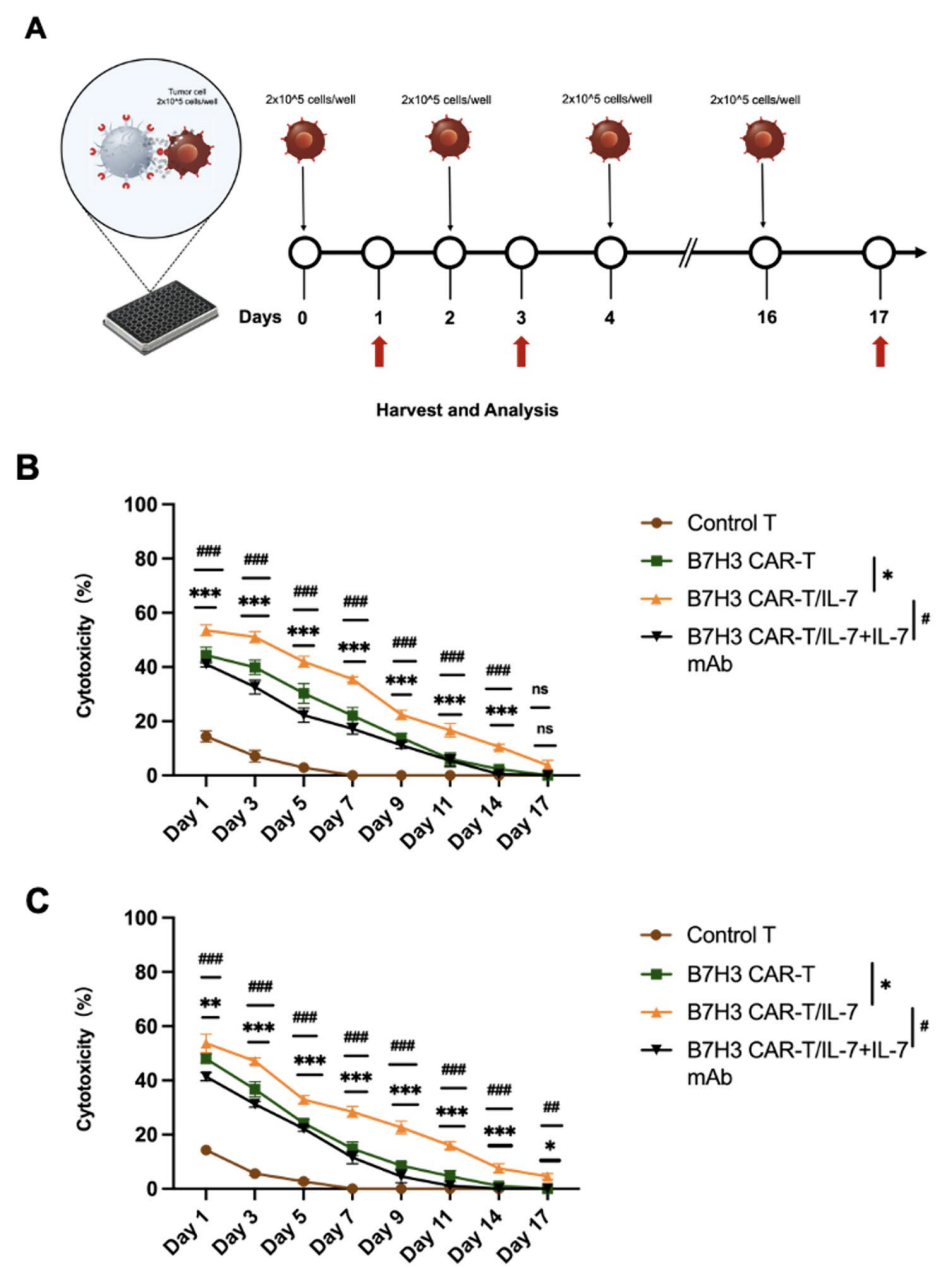
IL-7 improves the persistence of CAR-T cells during in vitro tumor re-challenges. **A** A schematic of the re-challenge experiment to assess CAR-T-cell persistence in vitro. **B** Sustained anti-tumor capacity of CAR-T cells against JHC-7 cells. **C** Sustained anti-tumor capacity of CAR-T cells against UCH2 cells. Data shown are mean ± SD (n = 3). *p < 0.05, **p < 0.01, ***p < 0.001, and ****p < 0.0001 (two-way ANOVA). Not significant (ns)

We next investigated IL-7-secreting CAR-T-cell behavior and efficacy against tumor cells using an in vitro long-term tumor challenge model. B7–H3 CAR-T and B7–H3 CAR-T/IL-7 cells were co-cultured with JHC-7 or UCH2 cells at an 8:1 ratio without cytokine support, with JHC-7 or UCH2 cells added to the co-culture system every 2 days. As control, B7–H3 CAR-T/IL-7 T cells with IL-7 mAb were treated in parallel (Fig. 4A). To evaluate the anti-tumor efficacy of CAR-T cells, we quantified LDH release every 3 days (Fig. 4B and C). Control T cells showed little cytotoxicity toward tumor cells and became largely ineffective by day 7. Conversely, B7–H3 CAR-T and B7–H3 CAR-T/IL-7 cells showed better anti-tumor persistence. In cytotoxicity assays against chordoma cells performed at different time points, B7–H3 CAR-T/IL-7 cells remained lethal to chordoma cells on day 17, and the addition of IL-7 mAb to the B7–H3 CAR-T/IL-7 and chordoma cell co-culture system attenuated the sustained killing of tumor cells.

### Regulation of *CAR*-T cells by IL-7

**Fig. 5:**
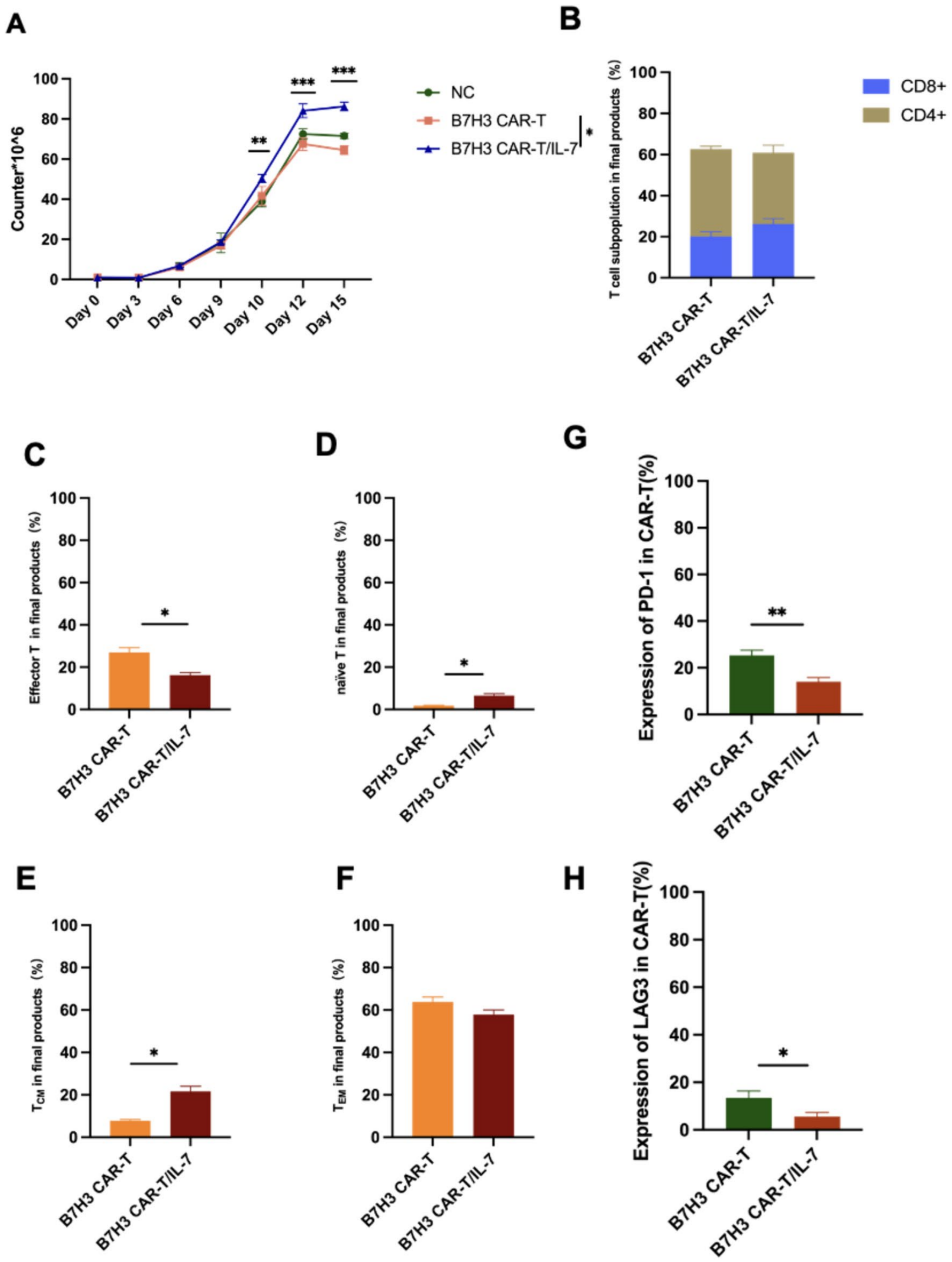
IL-7 regulates CAR-T-cell expression. **A** Expansion of T cells transduced with B7–H3 CAR and B7–H3 CAR/IL-7 cells. **B** Population assay of the CD8^+^ and CD4^+^ subpopulations in B7–H3 CAR-T or B7–H3 CAR-T/IL-7 cells after incubation for 12 days. **C–F** Subpopulations of memory T cells in the final B7–H3 CAR-T or B7H3 CAR-T/IL-7 cells were measured by flow cytometry on days 7–10. Subpopulations of different T cells were identified as follows: T_EM_, naïve T, effector T, and T_CM_. **G** PD-1 expression in B7–H3 CAR-T or B7–H3 CAR-T/IL-7 cells. **H** LAG-3 expression in B7–H3 CAR-T or B7–H3 CAR-T/IL-7 cells. Data shown are mean ± SD (n = 3). *p < 0.05, **p < 0.01, ***p < 0.001, and ****p < 0.0001 (two-way ANOVA). Not significant (ns)

To assess and contrast the in vitro expansion efficiencies of various B7–H3 CAR-T-cell structures, cells were cultured and quantified at multiple time points. B7–H3 CAR-T/IL-7 cells exhibited greater proliferation than B7–H3 CAR-T cells following a 12-day expansion period (Fig. 5A). Furthermore, flow cytometry analysis revealed that CD4^+^ B7–H3 CAR-T and B7–H3 CAR-T/IL-7 cells were more frequent than CD8^+^ B7–H3 CAR-T and B7–H3 CAR-T/IL-7 cells (Fig. 5B). The proportion of CD8^+^ B7–H3 CAR-T/IL-7 cells was higher than CD8^+^ B7–H3 CAR-T cells.

Subsequent analysis of the proportion of different T-cell subsets (effector T, naïve T, T_CM_, and T_EM_) in B7–H3 CAR-T cells (Fig. 5C, D, E, and F) revealed that the expression of naïve T cells and T_CM_ in B7–H3 CAR-T/IL-7 cells was higher than that in B7–H3 CAR-T cells. The proportion of effector T cells in B7–H3 CAR-T cells was higher than that in B7–H3 CAR-T/IL-7 cells. To examine the effect of IL-7 on T-cell immune checkpoint expression during T-cell culture, we quantified PD-1 and LAG-3 on the surface of cultured CAR-T cells (Fig. 5G and H), which showed greater expression on the surface of B7–H3 CAR-T cells than on B7–H3 CAR-T/IL-7 cells.

### B7–H3 *CAR*-T/IL-7 cells show enhanced therapeutic efficacy against primary chordoma cells

**Fig. 6:**
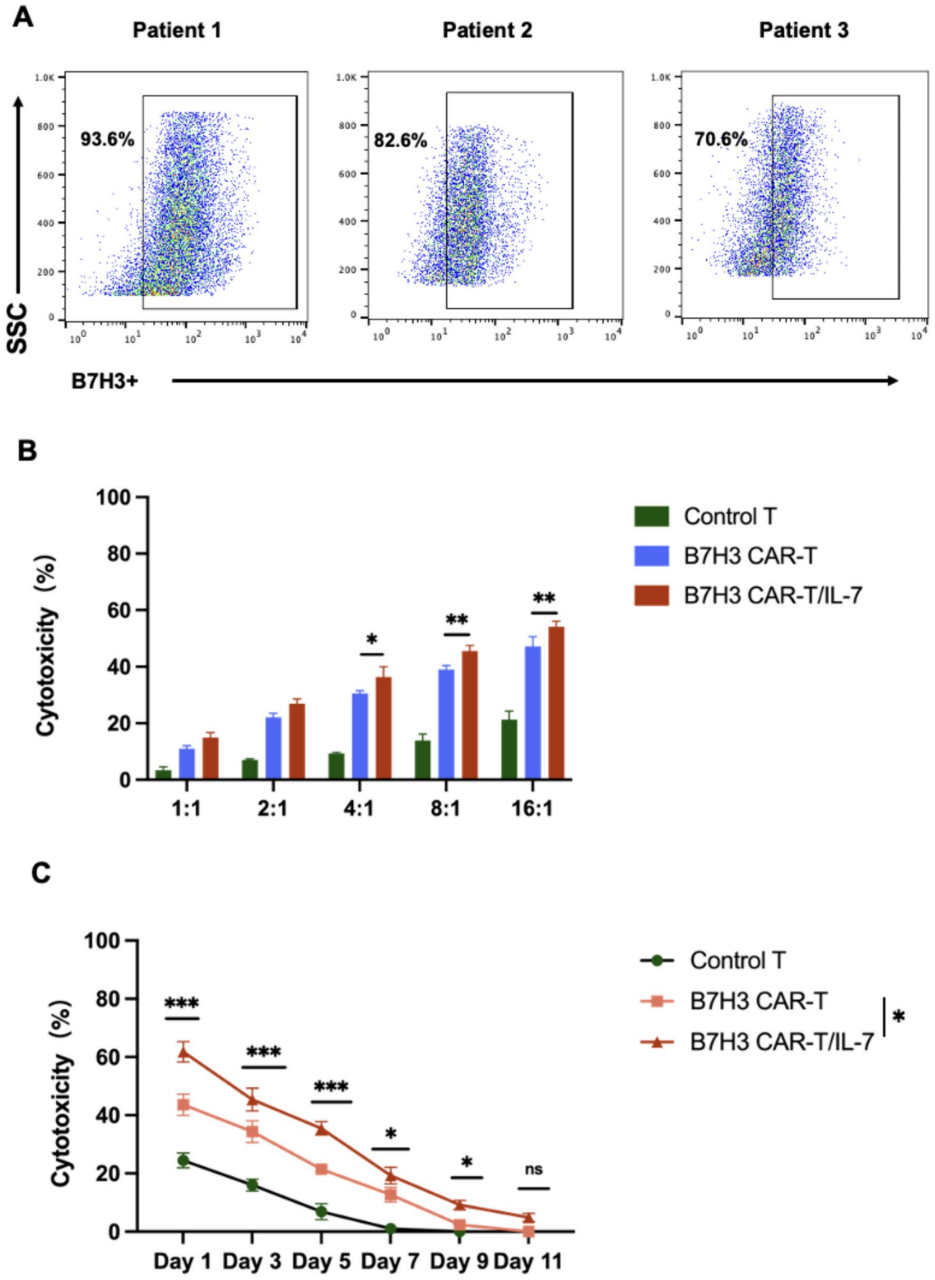
The therapeutic efficacy of B7–H3 CAR-T/IL-7 cells is superior to B7–H3 CAR-T cells when targeting primary chordoma cells. **A** Flow cytometry analysis showing B7–H3 expression in chordoma organoids derived from patients. **B** B7–H3 CAR-T and B7–H3 CAR-T/IL-7 cells were co-cultured with chordoma organoids derived from patients at different E/T ratios (from 0.5:1 to 8:1). Cytotoxicity was measured by the LDH release assay after a 20-h incubation. **C** B7–H3 CAR-T and B7–H3 CAR-T/IL-7 cells were co-cultured with multiple rounds of chordoma organoids. Long-term cytotoxicity was measured by the LDH release assay after 1–11 days of incubation. Data shown are mean ± SD (n = 3). *p < 0.05, **p < 0.01, ***p < 0.001, and ****p < 0.0001 (two-way ANOVA). Not significant (ns)

Finally, to evaluate the effectiveness of our CAR-T cells in the clinical context, we tested them in a patient-derived chordoma organoid model expressing B7–H3. B7–H3 was confirmed as highly expressed in chordoma organoid cells (Fig. 6A). Organoids were incubated with control, B7–H3 CAR-T, and B7–H3 CAR-T/IL-7 cells at different E/T ratios. B7–H3 CAR-T and B7–H3 CAR-T/IL-7 cells exhibited distinct cytotoxicity against organoids, with cytotoxicity positively associated with E/T ratios. The cytotoxicity of B7–H3 CAR-T/IL-7 cells was superior to that of B7–H3 CAR-T cells (Fig. 6B). To assess persistent cytotoxicity of CAR-T cells against primary chordoma cells, we developed a CAR-T-cell/primary chordoma cell co-culture system, with primary tumor cells introduced at intervals to evaluate sustained CAR-T-cell cytotoxicity. Notably, B7–H3 CAR-T/IL-7 cells exhibited superior sustained cytotoxicity toward chordoma organoids (Fig. 6C). Moreover, B7–H3 CAR-T cells effectively bound IL-7 within the system, mitigating immunosuppression and enhancing the cytotoxicity of CAR-T cells against chordoma cells.

## Discussion

Immunotherapy has emerged as one of the most promising therapeutic approaches in cancer patients [36, 37]. The aim of the present study was to identify new potential immunotherapy drug targets for chordoma. CAR-T therapy relies on the identification of a good tumor antigen as a target. An ideal therapeutic antigen would have homogeneous and specific overexpression in the tumor [38, 39]. B7–H3 is widely expressed in primary solid malignant tumors, with a low frequency of B7–H3 expression in hematological malignancies. For all cancers, the cumulative frequency of B7–H3 positivity is 59.5% [40–42]. Chordoma has also been reported to highly express B7–H3 [43]. Our sequencing results confirmed that B7–H3 is a highly expressed target in chordoma cells and could, therefore, be a target for chordoma therapy. In addition, expression of IL-7 in the patient’s tumor tissue and expression of the IL-7 receptor in T cells were higher than those of the control group, suggesting a T-cell-mediated immune response within the TME. We, therefore, speculated that CAR-T cells expressing IL-7 would further promote the immune response of T cells in the TME.

Although useful for the management of hematological malignancies, the application of CAR-T-cell therapy to solid tumors has been more difficult [44, 45]. These challenges primarily arise from the antigenic heterogeneity exhibited by cancer cells, the intricate composition of cellular elements within solid tumors, limited infiltration capacity and migration of CAR-T cells into tumor tissues, and the presence of an immunosuppressive TME [46]. Currently, CAR-T-cell therapy is predominantly administered as an autologous treatment, and its clinical effectiveness is closely related to the expansion capacity and persistence of CAR-T cells. Notably, there exist variations in the potency of CAR-T cells among individual patients, with a significant proportion exhibiting suboptimal T-cell function [29]. IL-7 plays a crucial role in promoting the survival of viable lymphocytes and is intricately involved in the development, persistence, and proliferation of T cells. The introduction of IL-7 has been shown to enhance the cytotoxicity of CAR-T cells against target cells in vivo. However, the limited half-life of IL-7 within the body poses a challenge to its therapeutic development, necessitating further research and optimization [47]. We, therefore, compared the cytotoxicity of two CAR-T-cell structures on chordoma cells and found that B7–H3 CAR-T/IL-7 had better cytotoxicity against chordoma cells. In a co-culture system, the cytotoxicity of B7–H3 CAR-T/IL-7 against chordoma cells was greater than that of B7–H3 CAR-T cells over time and, confirming the positive effect of Il-7, sustained tumor cytotoxicity by B7–H3 CAR-T/IL-7 cells decreased after addition of anti-IL-7 antibodies into the system. Furthermore, B7–H3 CAR-T/IL-7 cells showed better killing ability against patient-derived chordoma cells and sustained anti-tumor capacity.

In terms of underlying mechanisms, the cell proliferation efficiency of B7–H3 CAR-T/IL-7 cells was higher than that of B7H3 CAR-T cells, suggesting that IL-7 promotes T-cell proliferation. Further analysis of T-cell subsets showed that the proportion of CD8^+^ T cells in the B7–H3 CAR-T/IL-7 system was higher than that in the B7H3 CAR-T system. The proportion of naïve T and T_CM_ cells in B7–H3 CAR-T/IL-7 cells was also higher than that in B7H3 CAR-T cells. IL-7 would be expected to maintain T-cell stemness and, as a consequence, promote the cytotoxicity of B7–H3 CAR-T/IL-7 cells against tumor cells [30]. The high percentage of CD8^+^ T cells also promoted the killing ability of B7–H3 CAR-T/IL-7 cells against chordoma cells [48]. IL-7 maintains CAR-T cells in a less differentiated T-cell state, regulates metabolic activities, and prevents CAR-T-cell depletion, which may be critical for CAR-T cells to maintain their metabolic adaptations and anti-tumor responses for up to several years [47]. Immune checkpoint molecules play an important role in tumor immunosuppression [33], and CAR-T cells have reduced anti-tumor capacity during the treatment of solid tumors due to elevated expression of immune checkpoints in the tumor. We found that the expression of immune checkpoints PD-1 and LAG3 in B7–H3 CAR-T/IL-7 cells was lower than that in B7H3 CAR-T cells. IL-7 can increase cytotoxicity and enhance the anti-tumor effect of CAR-T cells by decreasing immune checkpoint expression in CAR-T cells [49–51].

In summary, we analyzed single-cell sequencing data from 14 chordoma patients and found that B7–H3 is highly expressed in chordoma patient samples, providing a potential target for chordoma treatment. We thus designed CAR-T cells targeting B7–H3 and, after finding that IL-7 is also highly expressed in chordoma samples, coupled sequences to secrete IL-7 from B7–H3 CAR-T cells, thereby enhancing the anti-tumor and sustained killing ability of CAR-T cells. B7–H3 CAR-T/IL-7 cells showed enhanced cytotoxicity and a sustained killing effect against chordoma cells. Further in vivo validation is now required to pave the way for clinical translation.

## Supplementary Information

Below is the link to the electronic supplementary material.

Supplementary file1 (DOCX 200 KB)

Supplementary file2 (XLSX 9 KB)
