# Supplementary material for: Interleukin-7 expression by CAR-T cells improves CAR-T cell survival and efficacy in chordoma
Source: Cancer Immunol Immunother. 2024 Aug 2;73(10):188. doi: 10.1007/s00262-024-03756-9 (PMC11297017; doi:10.1007/s00262-024-03756-9)

**SUPPLEMENTARY INFORMATION**

**Figure S1.** Top 10 differentially expressed genes in chordoma and nucleus pulposus samples.


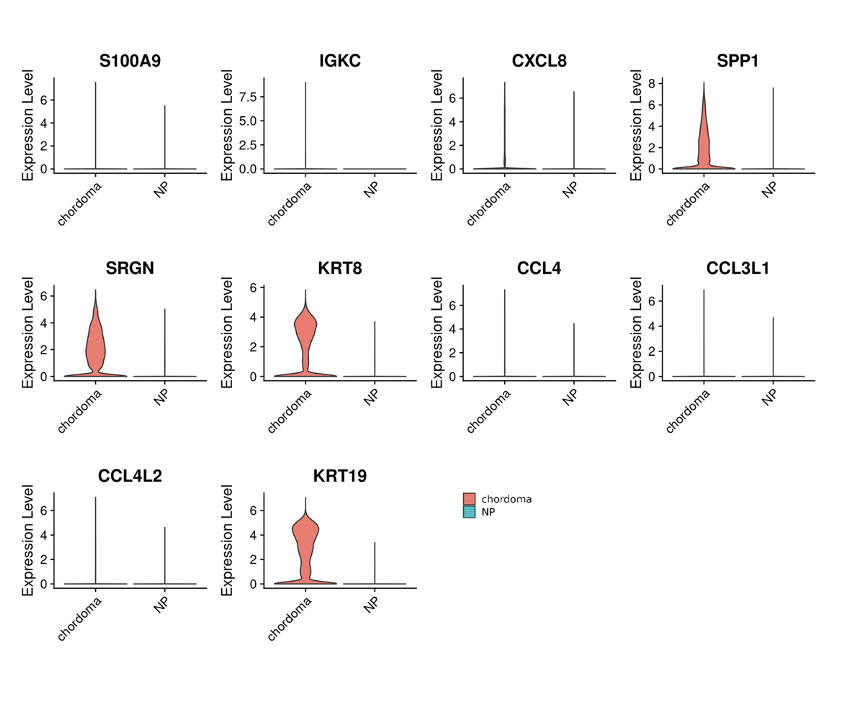


**Figure S2. B7-H3 CAR-T cells enhance anti-tumor cytotoxicity *in vitro.* (A)** B7-H3 CAR-T and B7-H3 CAR-T/IL-7 cells were co-cultured with UCH2, a chordoma cell line, at different E/T ratios (from 0.5:1 to 8:1). Cytotoxicity was measured by the LDH release assay after 20 h incubation. **(B-E)** ELISA data showing the quantification of cytokines (IL-6, IFN-γ, IL-2, and TNF-α) in the supernatants after B7-H3 CAR-T and B7-H3 CAR-T/IL-7 cells were co-cultured with UCH2 cells at an E:T of 8:1 for 20 h. Data shown are mean ± SD (n = 3). *p < 0.05, **p < 0.01, ***p <0.001, and ****p < 0.0001 (two-way ANOVA). Not significant (ns).


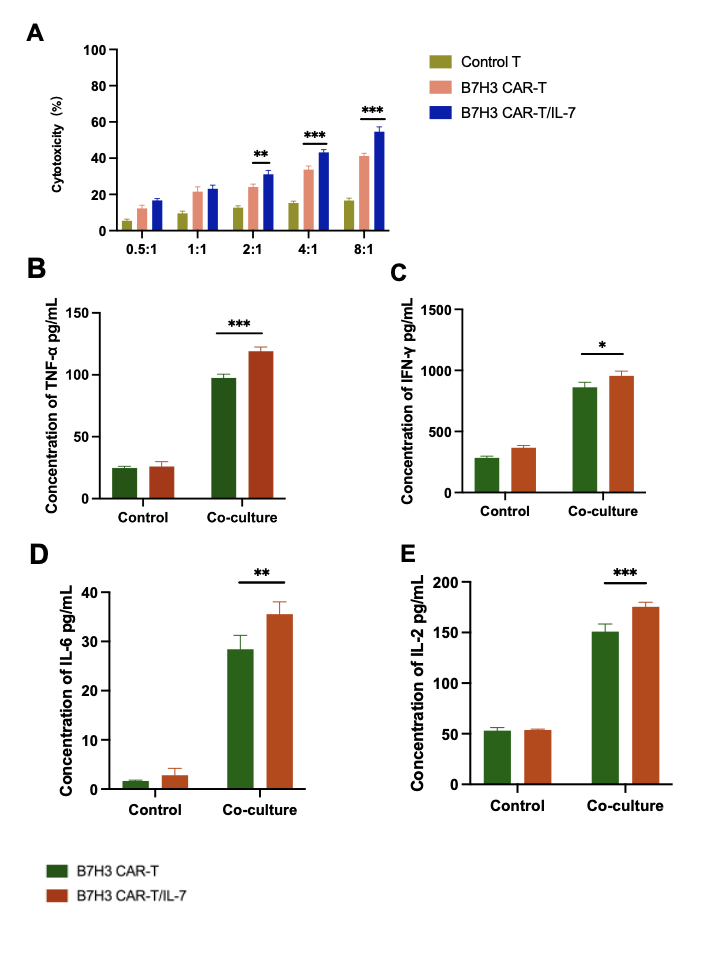

Supplement: Supplementary file 1 — Supplementary file1 (DOCX 200 KB) [file 262_2024_3756_MOESM1_ESM.docx]
